# Electronic cigarettes: emerging challenges in cessation and dependence management. A call for evidence-based guidelines to address a growing epidemic among Brazilian youth

**DOI:** 10.36416/1806-3756/e20250321

**Published:** 2025-09-22

**Authors:** Maria Enedina Claudino Aquino Scuarcialupi, Carlos Leonardo Carvalho Pessôa, Ramiro Dourado-Maranhão

**Affiliations:** 1. Afya Faculdade de Ciências Médicas da Paraíba - AFYA-Paraíba - Cabedello (PB) Brasil.; 2. Coordenação de Doenças Respiratórias, Centro de Referência Multiprofissional de Doenças Raras, Hospital Universitário Lauro Wanderley, Faculdade de Medicina, Universidade Federal da Paraíba - UFPB - João Pessoa (PB) Brasil.; 3. Comissão de Tabagismo, Sociedade Brasileira de Pneumologia e Tisiologia, Brasília (DF) Brasil.; 4. Disciplina de Pneumologia, Faculdade de Medicina, Universidade Federal Fluminense - UFF - Niterói (RJ) Brasil.; 5. Centro Universitário do Planalto Central Aparecido Santos - Uniceplac - Brasília (DF) Brasil.; 6. Fundação de Ensino e Pesquisa em Ciências da Saúde - FEPECS - Hospital Regional do Gama - Brasília (DF) Brasil.

The use of electronic cigarettes (vapes), a relatively recent form of nicotine consumption, has been consolidated into a global epidemic, with rising prevalence particularly among young people-including adolescents aged 13 to 24 years.^1^ Created by Chinese pharmacist Hon Lik in 2003,^2^ ^,^ ^3^ the device initially failed to gain widespread acceptance. The patent was later transferred to the tobacco industry, which heavily invested in marketing strategies emphasizing aesthetic appeal, a variety of flavors, attractive colors, innovative design, and the misleading claim that the product contained only water vapor and was harmless to health.^4^ ^-^ ^6^

These claims were swiftly refuted by scientific evidence and clinical experience, as reports have accumulated significant respiratory, cardiovascular, and mental health damage.^7^ ^-^ ^11^ In 2019, an outbreak of a previously undescribed pulmonary condition-E-cigarette or Vaping product use-Associated Lung Injury (EVALI)-was recorded, causing 68 deaths among young people in the United States, with cases and fatalities subsequently reported in other countries, including Brazil.^12^

In Brazil, the sales, transportation, and advertising of electronic cigarettes have been prohibited since 2009 by the Brazilian Health Regulatory Agency,^13^ a policy mirrored in countries such as Mexico, India, and Argentina. Conversely, nations such as Canada, the United States, and the United Kingdom have implemented additional restrictive measures, including raising the minimum purchase age, banning certain flavors, and limiting nicotine content.^6^ ^,^ ^14^ ^,^ ^15^

The appeal of electronic cigarettes has been heightened by the constant technological evolution of their devices. in 2024, data from the Brazilian national Telephone-based System for the Surveillance of Risk and Protective Factors for Chronic Diseases indicated that the average age of experimentation was around 13 years; that consumption was higher among boys; and that overall smoking prevalence increased from 9% in 2023 to 11.6% in 2024, possibly driven by vape use among youth.^16^ ^,^ ^17^ This scenario raises pressing questions: Has there been a relaxation in communication with the public and the medical community? Were there failures in enforcement and product seizures? Was there underestimation of the severity of the problem? Meanwhile, the tobacco industry’s lobbying power continues to influence the public and policymakers.

Structurally, e-cigarettes have four main components: a lithium battery, a tank, an atomizer, and a mouthpiece. Heating above 350°C produces an aerosol containing ultrafine particles and a complex mixture of chemicals-propylene glycol, glycerin, nitrites, heavy metals (such as lead and nickel),^8^ ^,^ ^10^ ^,^ ^18^ diacetyl, benzoic acid, and flavorings. Additionally, synthetic nicotine, nicotine salts, cannabis derivatives (CBD, THC), and amphetamines have been identified-all of which capable of inducing strong dependence-as shown in recent studies, including research conducted in Brazil by two important Brazilian universities.^18^ ^,^ ^19^

These devices primarily attract individuals who had never smoked, along with a smaller proportion of combustible cigarette smokers seeking, often mistakenly, to reduce health risks. However, national and international data show that dual use (conventional + electronic cigarettes) remains frequent and is increasing.^20^ ^,^ ^21^

The narrative review published by Martins et al.^22^ in this issue of the *Jornal Brasileiro de Pneumologia* provides health professionals with a broad approach to vape cessation, including behavioral support, nicotine replacement therapy (NRT), and non-nicotine pharmacological treatments. While this review is both relevant and timely, it is important to note that the scientific literature on vape cessation is still limited, frequently relying on studies with small sample sizes. Consequently, the review article featured in this issue makes a valuable contribution, highlighting the necessity for further research that specifies sample sizes and confidence intervals. On the other hand, it is important to stress that some studies referred in this review involve medications unavailable in Brazil, such as varenicline and cytisine, which could mislead clinical practice.

Another important aspect that warrants attention is that NRT has yet to be specifically validated for e-cigarette dependence. This is not unexpected, given the only recent rise in electronic cigarette use. However, caution is advised when applying the findings from NRT studies focused on traditional cigarette smokers to those who use electronic cigarettes.

Cognitive behavioral therapy, delivered individually or in groups, plays a central role in managing this population, composed mainly of young people undergoing physical and psychological maturation, with greater neuroplasticity and vulnerability to social influences.^14^ ^,^ ^23^ Cognitive-behavioral strategies that encourage reflection on self-image (“How do I see myself? How do I see the world? How do I think the world sees me?”), world perception, and social integration can help develop coping strategies for chemical and behavioral dependence. Complementary resources include physical, cultural, artistic, and manual activities, support apps, peer networks, and vape-free environments.

Applying theoretical models such as the stages of change,^24^ ^,^ ^25^ and validated instruments for dependence assessment (Penn State Nicotine Dependence Index, Modified Fagerström for e-cigs) can refine therapeutic planning. However, the high nicotine levels in vapes-often exceeding those in conventional cigarettes-pose challenges for simply adapting usual NRT regimens. Continuous NRT combined with rapid-release forms during peak cravings may be considered, always alongside intensive behavioral support.

It is important to note that the recommendations discussed here do not constitute formal guidelines, but rather provisional guidance. As with conventional smoking, pharmacological treatment in adolescents must be used with extreme caution and close follow-up, with behavioral therapy as the first-line approach.

To sum up, medical societies and health authorities must invest in continuing education, provide educational materials, and consolidate clinical protocols to ensure safe and effective professional practice in addressing e-cigarette dependence.
